# Mast cells dysregulate apoptotic and cell cycle genes in mucosal squamous cell carcinoma

**DOI:** 10.1186/1475-2867-6-28

**Published:** 2006-12-19

**Authors:** Sydney Ch'ng, Michael Sullivan, Lan Yuan, Paul Davis, Swee T Tan

**Affiliations:** 1 From the Wellington Regional Plastic, Maxillofacial & Burns Unit, Hutt Hospital, Wellington, New Zealand; 2 The Department of Medicine, Wellington School of Medicine & Health Sciences, Wellington, New Zealand; 3 The Children's Cancer Research Group, Christchurch School of Medicine & Health Sciences, Christchurch, New Zealand

## Abstract

**Background:**

Mucosal squamous cell carcinoma of the head and neck is a disease of high mortality and morbidity. Interactions between the squamous cell carcinoma and the host's local immunity, and how the latter contributes to the biological behavior of the tumor are unclear. *In vivo *studies have demonstrated sequential mast cell infiltration and degranulation during squamous cell carcinogenesis. The degree of mast cell activation correlates closely with distinct phases of hyperkeratosis, dysplasia, carcinoma *in-situ *and invasive carcinoma. However, the role of mast cells in carcinogenesis is unclear.

**Aim:**

This study explores the effects of mast cells on the proliferation and gene expression profile of mucosal squamous cell carcinoma using human mast cell line (HMC-1) and human glossal squamous cell carcinoma cell line (SCC25).

**Methods:**

HMC-1 and SCC25 were co-cultured in a two-compartment chamber, separated by a polycarbonate membrane. HMC-1 was stimulated to degranulate with calcium ionophore A23187. The experiments were done in quadruplicate. Negative controls were established where SCC25 were cultured alone without HMC-1. At 12, 24, 48 and 72 hours, proliferation and viability of SCC25 were assessed with MTT colorimetric assay. cDNA microarray was employed to study differential gene expression between co-cultured and control SCC25.

**Results:**

HMC-1/SCC25 co-culture resulted in suppression of growth rate for SCC-25 (34% compared with 110% for the control by 72 hours, p < 0.001), and dysregulation of genes TRAIL, BIRC4, CDK6, Cyclin G2 and CDC6 in SCC25.

**Conclusion:**

We show that mast cells have a direct inhibitory effect on the proliferation of mucosal squamous cell carcinoma *in vitro *by dysregulating key genes in apoptosis and cell cycle control.

## Background

Mucosal squamous cell carcinoma of the head and neck (HNSCC) is the sixth most common cancer afflicting men in the developed world. Despite diagnostic and therapeutic advances, there has been little improvement in the mortality over the last three decades [1]. Although the genetic events underlying HNSCC progression are slowly unravelling [2], very little is known about the interactions between the tumor and its microenvironement, more specifically the host's local immunity, and how the latter contributes to the biological behavior of the tumor. Among the immune cells (i.e., tumor-associated macrophages, dendritic cells, neutrophils, T cells and mast cells) in the microenvironment, mast cell has probably received the least attention despite well-established evidence for its roles in carcinogenesis [3].

Mast cells originate from the bone marrow and the immature progenitors migrate to peripheral tissues where they mature *in-situ*. Mast cells abound in intense peritumoral inflammation that often surrounds aggressive cancers, including melanoma, breast carcinoma and colorectal adenocarcinoma [4]. In laryngeal SCC specimens, mast cells are frequently present in clusters, whereas they scatter singly in the submucosa in healthy tissues [5]. Flynn *et al *demonstrate a direct correlation between sequential mast cell infiltration and activation, and distinct stages of hyperkeratosis, dysplasia, carcinoma *in-situ *and invasive squamous carcinoma in the oral cavity *in vivo *[6]. Mast cells have been implicated in conferring the angiogenic phenotype in pre-malignant lesions, and contributing to neovascularization during squamous epithelial carcinogenesis [7]. The effects of mast cells on carcinogenesis are likely to be mediated through multiple pathways, including immunosuppression, enhancement of angiogenesis, disruption of the extracellular matrix, and promotion of tumor cell mitosis [3].

Using MTT (3-[4,5-dimethylthiazole-2-yl]-2,5-diphenyl tetrazolium bromide) cell proliferation and cDNA microarray assays, we investigated the effects of a human mast cell line co-culture on the rate of proliferation, and gene expression profile of a human tongue squamous cell carcinoma (SCC) cell line. We demonstrated mast cell-mediated cytotoxicity, and dysregulation of apoptotic and cell cycle genes in the mucosal SCC cell line.

## Results

**Figure 1:**
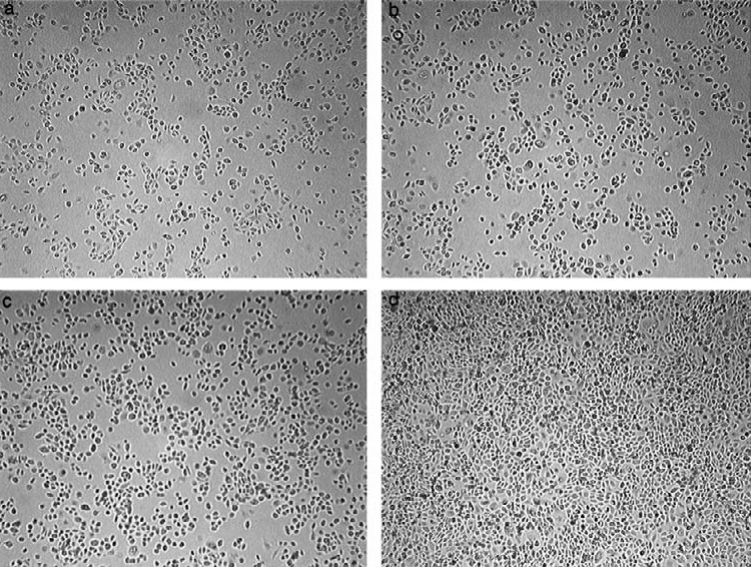
SCC25 co-cultured with (a) HMC-1 and (b) control SCC25 at 12 hours did not show discernible growth difference on microscopic examination. At 72 hours, (d) control SCC25 had outgrown (c) co-cultured SCC25, forming a confluent monolayer on the lower compartment of the culture plate.

**Figure 2:**
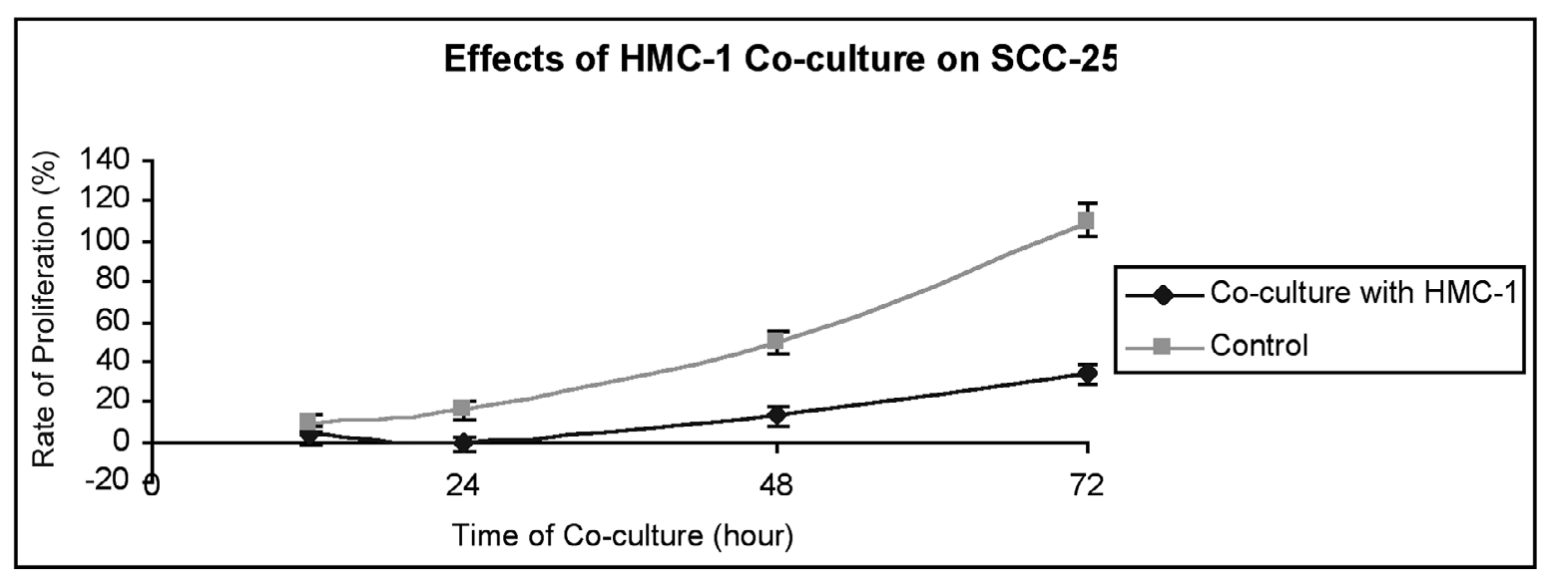
Rate of proliferation of SCC25 as determined by MTT colorimetric assay. Co-culture of HMC-1 with SCC25 caused a decreased rate of proliferation evident by 12 hours, and the discrepancy in proliferation continued to increase at 24, 48 and 72 hours.

HMC-1/SCC25 co-culture resulted in suppression of growth rate for SCC25, which became statistically significant by 24 hours (p < 0.001) (Figures 1 and 2). The growth discrepancy increased with time, and by 72 hours the growth rate of co-cultured SCC25 was less than a third that of the control (34% and 110% respectively). At 24 hours, the co-cultured SCC25 cell count dipped below the starting value (Figure 2), although the rate of proliferation improved subsequently. Morphology differences in the co-cultured SCC25 cells were obvious as compared to the control. Co-culture with HMC-1 resulted in considerably more rounded and shrunken SCC25 cells, many of which subsequently became non-viable and detached from the confluent monolayer.

**Figure 3:**
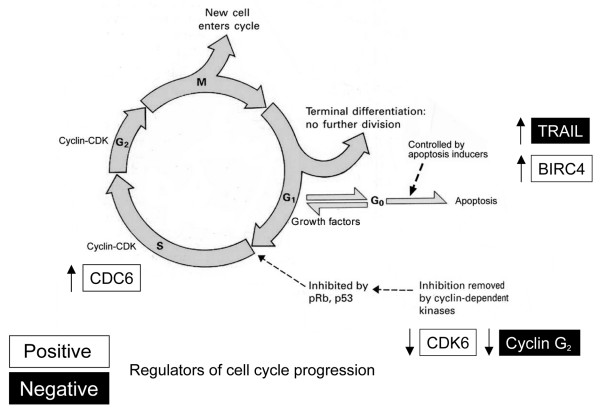
Schematic representation of dysregulation of apoptotic and cell cycle genes in SCC25 when co-cultured with HMC-1.

HMC-1 affected the gene expression profile of SCC25 in co-culture (Figure 3) (Table 1). Two members of the apoptosis panel were found to be significantly up-regulated, tumor necrosis factor-related apoptosis-related ligand (TRAIL) and baculoviral inhibitor of apoptosis repeat-containing protein 4 (BIRC4). Several candidate genes known to play a role in cell division were found to be dysregulated, including up-regulation of cell division cycle 6 (CDC6), and down-regulation of cyclin-dependent kinase 6 (CDK6) and cyclin G2 (CCNG2).

**Table 1:** Dysregulated genes in SCC25 after co-culture with HMC-1.

Accession no.	Gene name	Average fold change	*p *value
NM_003810	TRAIL/TNFSF10	1.6	0.01
NM_001167	BIRC4	1.7	0.02
NM_001254	CDC6	2.1	0.01
NM_004354	CCNG2	0.5	0.02
NM_001259	CDK6	0.5	0.04

## Discussion

Like other inflammatory cells, mast cells are attracted to tumors by various factors, including hypoxia, cellular damage, tissue ischemia and tumor-derived chemoattractants, including stem cell factor, interleukins-3 (IL-3) and IL-4 [8]. They in turn produce various cytokines, such as tumor necrosis factor-α (TNF-α), IL-1, IL-4 and IL-6, which can induce apoptosis of tumor cells. Mast cells are also known to stimulate anti-tumor lymphocytes through IL-8 and RANTES [9]. The cytokines produced by mast cells effecting genetic changes in the target cells, however, are beyond the scope of this study.

### Apoptosis panel

Our study shows that TRAIL is up-regulated in the co-cultured SCC-25 target cells, possibly through mast cell-derived interferon-γ (IFN-γ). Up-regulation of TRAIL gene expression and protein synthesis is known to occur in Ewing sarcoma, and thyroid carcinoma cell lines following intereferon (IFN)/cytokine treatment, contributing to apoptosis of the malignant cells in an autocrine and paracrine manner [10,11]. TRAIL serum levels in melanoma patients are found to be significantly elevated following IFN-α administration [12]. The protein encoded by this gene is a cytokine that belongs to the TNF ligand family [13]. It is an immunological apoptotic inducer that preferentially kills virus-infected, transformed and tumor cells, but spares normal cells [13,14]. The binding of TRAIL to its receptors triggers activation of MAPK8/JNK, caspase 8 and caspase 3 [13]. Recent finding that TRAIL induces apoptosis in endothelial cells suggests that it may have an indirect anti-angiogenesis property in addition to its tumor cytotoxic effect [15]. Preclinical experiments have demonstrated the efficiency of recombinant human TRAIL and monoclonal antibody against TRAILR1 and TRAILR2 on human breast, colon, and uterine cancers [16].

BIRC4, an endogenous apoptosis inhibitor, is found to be up-regulated. The protein encoded by BIRC4 belongs to a family of highly conserved apoptosis suppressor proteins that bind to TNF receptor-associated factors, TRAF1 and TRAF2. It inhibits apoptosis induced by menadione, caspase 3 and caspase 7 [13].

### Cell cycle panel

HMC-1 down-regulates CDK6 expression in SCC25. CDKs are important regulators of cell cycle progression. CDK6, which first appears in mid-G1 phase, is important for G1 phase progression and G1/S transition. Together with CDK4, they regulate the activity of Retinoblastoma (Rb) tumor suppressor protein [13]. Exit from the G1 phase of the cell division cycle is regulated by phosphorylation of pRb by cyclin D/CDK4 and cyclin D/CDK6 complexes. Dysregulation of these critical kinases causes pRb inactivation resulting in deregulation of the cell cycle control. Increased expression of CDK6 has been shown as a mechanism for Rb inactivation in oral SCC [17]. Recently, the growth of melanoma cell lines has been successfully retarded *in vitro *by down-regulating their CDK6 gene expression with small interfering RNA [18]. Down-regulation of CDK6, coupled with reciprocal up-regulation of Rb (unpublished data, fold change 1.9, p = 0.09) will result in suppression of cell growth as observed in this study.

In addition to up-regulation of BIRC4 and CDC6, and down-regulation of CCNG2 contradict our finding of HMC-1 exerting an overall inhibitory effect on SCC25 proliferation. The DNA replication licensing protein encoded by CDC6 assembles to form one of the pre-replicative complexes required for DNA replication. CDC6 is over-expressed in dysplastic cells. CDC6 mRNA expression increases in a linear fashion in cervical squamous carcinogenesis, from normal cervix through cervical intraepithelial neoplasia to invasive cervical carcinoma [19].

CCNG2 has been suggested as a negative regulator of cell cycle progression. Its dysregulation is implicated in epithelial transformation and the early stages of human oral cancer development. Transfection of human oral SCC cell line with CCNG2 induces cell arrest in the G1 phase, resulting in a significant inhibition of cellular proliferation [20].

As some mast cells secrete a myriad of mediators, many with opposing effects, it is not surprising that genes that are known to inhibit and promote tumor proliferation are both dysregulated. The net effect on growth undoubtedly depends on an intricate interplay between these genes. Our study demonstrates an overall inhibitory effect of mast cells on the proliferation of HNSCC.

Our co-culture study represents the simplest of models to study the effects of mast cells on HNSCC, in a one-to-one relationship. In reality, mast cells are among a very heterogenous population of cells in the tumor microenvironment [4,8]. They are exposed to a multitude of signals with different temporal patterns from the tumor cells, non-malignant resident cells (e.g., fibroblasts) and other inflammatory/immune cells (e.g., macrophages).

Our study has failed to show an unequivocal direction in the change of gene expression in the target cells, reinforcing the screening nature of microarray tests. Validation of the proposed genes with RT-PCR, and if confirmed, follow-up investigation of potential mast cell-derived mediators effecting change in the target cells e.g., TNF-α, IL-1, IL-4 and IL-6 by siRNA gene knock-down study or subjecting the co-culture supernatant to Western-blotting would be a logical approach.

Given the plasticity and versatility of mast cells, their phenotype may change, being inhibitory or stimulatory to tumor development, depending on the microenvironment [21]. It is also possible that mast cells are initially recruited to the tumors as part of the host defence system, but subsequently become enmeshed within the stroma participating in carcinogenesis [21].

The challenge to targeting mast cells in cancer treatment therefore lies in selective inhibition of tumor-promoting mediators while sparing cytotoxic ones, or identifying and blocking causative factors contributing to their unfavorable phenotypic alteration.

## Conclusion

Mast cells inhibit HNSCC proliferation *in vitro *by dysregulating apoptotic and cell cycle gene expression.

## Materials and methods

### Cell lines

Human mast cell line (HMC-1) was obtained from Dr J. Butterfield (Mayo Clinic, MN, USA) and grown in Iscove's medium (with 25 mM HEPES, sodium bicarbonate, L-glutamine; without alpha thioglycerol or beta mercaptoethanol) (Gibco, NY, USA), 10% iron-supplemented bovine calf serum (Hyclone, Auckland, NZ) and 1.2 mM alpha thioglycerol (Sigma, MO, USA) at 37°C and 5% CO_2_. SCC25 was obtained from American Type Culture Collection and maintained in 1:1 mixture of DMEM and Ham's F12 medium (with 1.2 g/L sodium bicarbonate, 2.5 ml L-glutamine, 15 mM HEPES and 0.5 mM sodium pyruvate supplemented with 400 ng/ml hydrocortisone) (Gibco, NY, USA) and 10% fetal bovine serum (Hyclone, Auckland, NZ) at 37°C and 5% CO_2_. For the purpose of the co-culture experiment, SCC25 was subsequently harvested by trypsinization and cultured in fresh Iscove's medium as described. This did not impact negatively on the viability as determined by tryptan blue exclusion test.

### Co-culture of SCC25 and HMC-1

**Figure 4:**
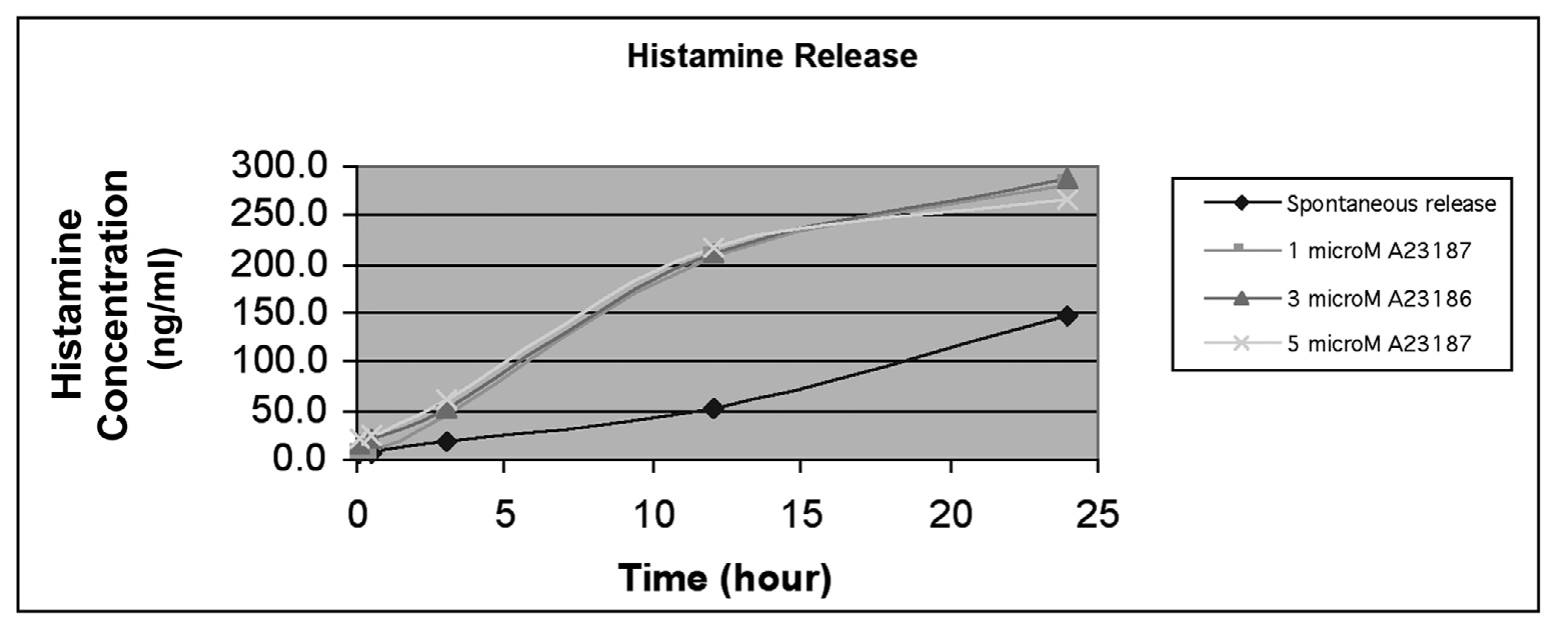
Optimal degranulation of HMC-1 with calcium ionophore A23187 was achieved with 1 μM concentration. Degranulation plateaued at 12 hours.

SCC25 at a cell density of 3.5 × 10^4^/ml (×2.5 ml) was cultured in the lower compartment of a 35-mm co-culture well (Corning, MA, USA). 5.8 × 10^4^/ml of HMC-1 (×1.5 ml) was seeded on the polyester transwell filter (pore size 0.4 μm) (Corning, MA, USA) in the upper compartment, giving an HMC-1:SCC25 co-culture ratio of 1:1. The cells were co-cultured in Iscove's medium as specified above. HMC-1 were then degranulated with 1 μM calcium ionophore A23187 (Sigma, MO, USA). The cells were co-cultured for 72 hours. Negative controls were established in which SCC25 were cultured alone without HMC-1, and A23187 was added to the empty upper compartment of the co-culture chambers. Culture medium was renewed every 24 hours. Histamine release was adopted as a surrogate marker for measurement of mast cell degranulation [22]. 1 μM A23187 was used as secretagogue in this study as higher concentrations did not show further increase in histamine release (Figure 4), and at 1 μM, A23187 did not affect viability of cultured cells as determined by tryptan blue exclusion test. Degranulation of HMC-1 was shown to plateau at 12 hours after stimulation (Figure 4).

### Cell proliferation assay

Co-culture experiments were conducted for 12, 24, 48 and 72 hours, at the end of which SCC25 were subjected to colorimetric quantification of proliferation and viability with MTT assay (Sigma, MO, USA). Four sets of co-culture experiments were performed for each time frame (n = 4).

Data for the co-culture experiments was subjected to analysis of variants with terms for co-culture effect, time and the interaction between co-culture effect and time, done with the log of the data, and with a random term for the culture wells.

### cDNA microarray analysis

#### RNA extraction and cDNA synthesis

Six sets of co-culture experiments and negative controls were carried out. The upper compartment of the co-culture chambers containing HMC-1 was discarded at 72 hours. SCC25 cells were lysed directly in their culture wells with Trizol (Sigma, MO, USA), and total RNA extracted as per manufacturer's instructions. The total RNA isolated was pooled. The quantity, purity and integrity of the RNA samples were assessed with spectrophotometer and gel electrophoeresis.

Reference RNA pooled from 14 human cancer cell lines was available upon request (Pacific Edge Biotechnology, Dunedin, NZ). cDNA synthesis from SCC25 and reference RNA was carried out with SuperScript III Reverse Transcriptase (Invitrogen, CA, USA) as per protocol.

#### cDNA labeling and microarray hybridization

SCC25 and reference cDNA samples were labeled with DMSO-suspended Molecular Probes, Alexa Fluor 647 and Alexa Fluor 555 (Invitrogen, CA, USA), respectively. The cDNA samples were then purified with the QIAquick PCR Purification Kit (Qiagen, CA, USA).

Genome-scale (30 000), oligonucleotide microarray slide (Pacific Edge Biotechnology, Dunedin, NZ) post-processing, pre-washing, and hybridization were performed according to the manufacturer's recommendations.

#### Data filtering and analysis

Signal intensity and analysis were determined using GenePixPro 4.0 Array Acquisition and Analysis Software for GenePix 4000B. Ratio count for the two wavelengths on the histogram was scaled to 0.95 – 1.05. Signal intensities were taken as "foreground median – background", and were excluded if they were below 50. Any spot less than 10 nm in size and that was not present on all slides was excluded. Each array was normalized using the lowess smoother method. The filter for gene expression fold change (up-regulation and down-regulation) was set at ≥ 1.5. 6 525 genes fulfilled these criteria.

Data analysis was performed with Biometric Research Branch – Array Tools 3.1.0. SCC25 co-cultured and control samples were subjected to class comparison and two-sample *t*-test. A *p*-value of < 0.05 was taken to indicate statistical significance. 458 of the 6 525 genes satisfied this criterion.

These 458 genes were analysed against known gene ontologies of tumorigenesis, metastasis, apoptosis and cell cycle as profiled in BioRag (Bioresource for Array Genes) [23], PubMatrix [13] and Source Database, Stanford Genomic Resources [24], totalling 250 individual genes [see Additional file 1]. 5 genes matched. Further analysis on remaining genes forms the basis of another ongoing study.

## Supplementary Material

Additional file 1 Genes known to feature in tumorigenesis, metastasis, apoptosis and cell cycle control gene ontologies. This table lists 250 genes that are known to be involved in tumorigenesis, metastasis, apoptosis and cell cycle control. Click here for file
