# Supplementary material for: Mast cells dysregulate apoptotic and cell cycle genes in mucosal squamous cell carcinoma
Source: Cancer Cell Int. 2006 Dec 19;6:28. doi: 10.1186/1475-2867-6-28 (PMC1769399; doi:10.1186/1475-2867-6-28)
Supplement: Additional file 1 — Genes known to feature in tumorigenesis, metastasis, apoptosis and cell cycle control gene ontologies. This table lists 250 genes that are known to be involved in tumorigenesis, metastasis, apoptosis and cell cycle control. [file 1475-2867-6-28-S1.doc]

**Supplement 1**

Genes known to feature in tumorigenesis, metastasis, apoptosis and cell cycle control gene ontologies

Accession no. Gene name

| NM007313 | ABL1 |
| --- | --- |
| NM001160 | APAF1 |
| NM006595 | API5 (apoptosis inhibitor 5) |
| NM013258 | ASC |
| NM000051 | ATM |
| NM004323 | BAG1 |
| NM004281 | BAG3 |
| NM004874 | BAG4 |
| NM001188 | BAK1 |
| NM004656 | BAP1 |
| NM004324 | BAX |
| NM014417 | BBC3 |
| NM003921 | BCL10 |
| NM000633 | BCL2 |
| NM004049 | BCL2A1 |
| NM006538 | BCL2L11 |
| NM052842 | BCL2L2 |
| NM016561 | BFAR |
| NM001197 | BIK |
| NM004536 | BIRC1 |
| NM001166 | BIRC2 |
| NM001165 | BIRC3 |
| NM001167 | BIRC4 |
| NM001168 | BIRC5 |
| NM016252 | BIRC6 |
| NM001715 | BLK |
| NM004052 | BNIP3 |
| NM032515 | BOK |
| NM007294 | BRCA1 |
| NM015399 | BRMS1 (BrMS1) |
| NM033293 | CASP1 |
| NM032974 | CASP10 |
| NM012114 | CASP14 |
| NM032982 | CASP2 |
| NM032991 | CASP3 |
| NM001225 | CASP4 |
| NM004347 | CASP5 |
| NM032992 | CASP6 |
| NM001227 | CASP7 |
| NM033355 | CASP8 |
| NM012115 | CASP8AP2 |
| NM032996 | CASP9 |
| NM001753 | CAV1 (caveolin-1) |
| NM003914 | CCNA1 |
| NM001237 | CCNA2 |
| NM031966 | CCNB1 |
| NM004701 | CCNB2 |
| NM005190 | CCNC |
| NM053056 | CCND1 |
| NM001759 | CCND2 |
| NM001238 | CCNE1 |
| NM004702 | CCNE2 |
| NM001761 | CCNF |
| NM004060 | CCNG1 |
| NM004354 | CCNG2 |
| NM001239 | CCNH |
| NM000610 | CD44 |
| NM003903 | CDC16 |
| NM001786 | CDC2 |
| NM001255 | CDC20 |
| NM001789 | CDC25A |
| NM021872 | CDC25B |
| NM001790 | CDC25C |
| NM001256 | CDC27 |
| NM004359 | CDC34 |
| NM007065 | CDC37 |
| NM003504 | CDC45L |
| NM001254 | CDC6 |
| NM003503 | CDC7 |
| NM004360 | CDH1 (cadherin-1/ E-cadherin) |
| NM052827 | CDK2 |
| NM000075 | CDK4 |
| NM001259 | CDK6 |
| NM001799 | CDK7 |
| NM001260 | CDK8 |
| NM078467 | CDKN1A |
| NM004064 | CDKN1B |
| NM000076 | CDKN1C |
| NM058195 | CDKN2A |
| NM004936 | CDKN2B |
| NM001262 | CDKN2C |
| NM007942 | CDKN2D |
| NM003879 | CFLAR |
| NM001274 | CHEK1 |
| NM007194 | CHEK2 |
| NM001279 | CIDEA |
| NM014430 | CIDEB |
| NM001827 | CKS2 |
| NM001846 | COL4A2 (collagen a2(IV)) |
| NM003805 | CRADD |
| NM000757 | CSF1 |
| NM005211 | CSF1R(c-fms/MC-SF-R) |
| NM000099 | CST3 (cystatin C) |
| NM001908 | CTSB (cathepsin B) |
| NM001909 | CTSD (cathepsin D) |
| NM001912 | CTSL (cathepsin L) |
| NM003592 | CUL1 |
| NM003591 | CUL2 |
| NM003590 | CUL3 |
| NM003589 | CUL4A |
| NM003588 | CUL4B |
| NM003478 | CUL5 |
| NM014326 | DAPK2 |
| NM005215 | DCC |
| NM004401 | DFFA |
| NM004402 | DFFB |
| NM005225 | E2F1 |
| NM004091 | E2F2 |
| NM001949 | E2F3 |
| NM001950 | E2F4 |
| NM001951 | E2F5 |
| NM001952 | E2F6 |
| NM001972 | ELA2 (elastase) |
| NM006209 | ENPP2 (autotaxin/ ATX) |
| NM004448 | ERBB2 (c-erb-2/ neu) |
| NM005238 | ETS1 (c-ets-1) |
| NM005239 | ETS2 (c-ets-2) |
| NM001986 | ETV4 (PEA3) |
| NM003824 | FADD |
| NM002005 | FES |
| NM000800 | FGF1 (aFGF) |
| NM002006 | FGF2 (bFGF) |
| NM005252 | FOS (c-fos) |
| NM001924 | GADD45A |
| NM000601 | HGF (Scatter factor) |
| NM006665 | HPSE (heparanase) |
| NM005343 | HRAS (c-hRas) |
| NM003806 | HRK |
| NM004507 | HUS1 |
| NM003259 | ICAM5 (telencephalin) |
| NM000612 | IGF2 |
| NM002203 | ITGA2 |
| NM002204 | ITGA3 |
| NM002205 | ITGA5 |
| NM000210 | ITGA6 |
| NM002211 | ITGB1 |
| NM000212 | ITGB3 |
| NM002231 | KAI1 |
| NM002256 | KISS1 (KiSS-1) |
| NM002291 | LAMB1 (laminin b1) |
| NM002293 | LAMC1 (laminin b2) |
| NM002314 | LIMK1 (LIM kinase) |
| NM000595 | LTA |
| NM002341 | LTB |
| NM002342 | LTBR |
| NM002358 | MAD2L1 |
| NM006341 | MAD2L2 |
| NM003010 | MAP2K4 (mkk4 (JNKK1)) |
| NM021960 | MCL1 |
| NM004526 | MCM2 |
| NM002388 | MCM3 |
| NM006739 | MCM5 |
| NM005915 | MCM6 |
| NM005916 | MCM7 |
| NM002392 | MDM2 |
| NM002409 | MGAT3  (acetylglucosaminyltransferase 3) |
| NM002410 | MGAT5  (acetylglucosaminyltransferase 5) |
| NM012215 | MGEA5  (meningioma hyaluronidase 5) |
| NM000247 | MICA (MUC-18) |
| NM002417 | MKI67 |
| NM002421 | MMP1 |
| NM002425 | MMP10 |
| NM005940 | MMP11 |
| NM002427 | MMP13 |
| NM004995 | MMP14 |
| NM002428 | MMP15 |
| NM005941 | MMP16 |
| NM004530 | MMP2 |
| NM002422 | MMP3 |
| NM002423 | MMP7 |
| NM002424 | MMP8 |
| NM004994 | MMP9 |
| NM005591 | MRE11A |
| NM004689 | MTA1 |
| NM002456 | MUC1 |
| NM002467 | MYC (c-myc) |
| NM002468 | MYD88 |
| NM002485 | NBS1 |
| NM000615 | NCAM1 |
| NM006156 | NEDD8 |
| NM002506 | NGFB |
| NM005009 | NME4 |
| NM002539 | ODC1 |
| NM002592 | PCNA |
| NM000442 | PECAM1 |
| NM002646 | PIK3C2B |
| NM002658 | PLAU (uPA) |
| NM002659 | PLAUR (uPAR) |
| NM003981 | PRC1 |
| NM000314 | PTEN |
| NM000963 | PTGS2 (cox-2) |
| NM006908 | RAC1 |
| NM002873 | RAD17 |
| NM005732 | RAD50 |
| NM002875 | RAD51 |
| NM002880 | RAF1 |
| NM000321 | RB1 |
| NM002895 | RBL1 |
| NM005611 | RBL2 |
| NM014248 | RBX1 |
| NM003804 | RIPK1 |
| NM003821 | RIPK2 |
| NM002947 | RPA3 |
| NM002575 | SERPINB2 (PAI-2) |
| NM002639 | SERPINB5 (maspin) |
| NM000602 | SERPINE1 (PAI-1) |
| NM005983 | SKP2 |
| NM003087 | SNCG (BCSG1) |
| NM000582 | SPP1 (osteopontin) |
| NM005417 | SRC (c-src) |
| NM004180 | TANK |
| NM007111 | TFDP1 |
| NM006286 | TFDP2 |
| NM003236 | TGFA (TGF-a) |
| NM000660 | TGFB1 (TGF-b1) |
| NM003246 | THBS1 |
| NM003247 | THBS2 |
| NM003254 | TIMP1 |
| NM003255 | TIMP2 |
| NM000362 | TIMP3 |
| NM019894 | TMPRSS4 |
| NM000594 | TNF |
| NM003844 | TNFRSF10A |
| NM003842 | TNFRSF10B |
| NM002546 | TNFRSF11B |
| NM003820 | TNFRSF14 |
| NM001192 | TNFRSF17 |
| NM004195 | TNFRSF18 |
| NM001065 | TNFRSF1A |
| NM001066 | TNFRSF1B |
| NM014452 | TNFRSF21 |
| NM003327 | TNFRSF4 |
| NM001250 | TNFRSF5 |
| NM000043 | TNFRSF6 |
| NM032945 | TNFRSF6B |
| NM001242 | TNFRSF7 |
| NM001243 | TNFRSF8 |
| NM001561 | TNFRSF9 |
| NM003810 | TNFSF10 |
| NM003808 | TNFSF13 |
| NM006573 | TNFSF13B |
| NM003807 | TNFSF14 |
| NM005118 | TNFSF15 |
| NM005092 | TNFSF18 |
| NM003326 | TNFSF4 |
| NM000074 | TNFSF5 |
| NM000639 | TNFSF6 |
| NM001252 | TNFSF7 |
| NM001244 | TNFSF8 |
| NM003811 | TNFSF9 |
| NM000546 | TP53 |
| NM003722 | TP73L |
| NM005658 | TRAF1 |
| NM021138 | TRAF2 |
| NM003300 | TRAF3 |
| NM004295 | TRAF4 |
| NM004619 | TRAF5 |
| NM004620 | TRAF6 |
| NM005879 | TRIP |
| NM003334 | UBE1 |
| NM000462 | UBE3A |
| NM003352 | UBL1 |
| NM003376 | VEGF |
| NM005429 | VEGFC |
| NM000638 | VTN (vitronectin) |
